# Homophily in coauthorship networks of East European sociologists

**DOI:** 10.1038/srep36152

**Published:** 2016-10-27

**Authors:** Marian-Gabriel Hâncean, Matjaž Perc

**Affiliations:** 1 The Research Institute of the University of Bucharest & Department of Sociology, University of Bucharest, Schitu Măgureanu, 9, 010181, Bucharest, Romania; 2 Faculty of Natural Sciences and Mathematics, University of Maribor, Koroška cesta 160, SI-2000, Maribor, Slovenia

## Abstract

We study to what degree and how homophily and network properties affect individual citation counts of researchers in the sociology departments of three East European countries, namely Poland, Romania, and Slovenia. We built first-order personal coauthorship networks out of the Web of Science publication records. Each sociologist is assigned as a focal node or ego, while her coauthors are alters. We analyze the data using structural measurements methods, hierarchical regression models, and we make visualizations based on the clustered graph technique. For all three populations, our results indicate that the mean score of the citations of alters substantially predicts the citation counts of egos. In particular, citation similarity increases the chances for coauthorship ties. Evidence for the impact of network properties on the citation levels of egos is mixed. For Poland, normalized ego-betweenness shows a negative effect on citation counts, while network density displays a positive one. For Romania and Slovenia, network characteristics have only a minor impact. Even if the visual summarization of the personal networks uncovers a wide palette of coauthorship patterns, homophily appears to be pervasive. These results are relevant for domestic policy makers who aim to improve the aggregated research performance in East European countries.

Modern social network analysis, generally, builds on the assumption that network patterns have significant consequences for the embedded actors^1^. Actors’ outcomes (both benefits and disadvantages) and future characteristics are heavily affected, not only by the actor’s own attributes, but also by the structural properties of their network positions^2^. During the last decades, social network analysis has become extremely popular, being applied in a variety of disciplines, such as social sciences, physics, epidemiology, biology^3^, informetrics^4^, scientometrics^5^ and bibliometrics^6^.

The application of social network analysis to the study of coauthorship activity^7^,^8^,^9^,^10^,^11^,^12^ has proved to be extremely fruitful in understanding coauthorship tie creation, the diffusion of innovation, the academic success and career longevity, the creation of prestige, the increase of visibility, the intellectual structure in science, the existence of research fronts etc. Commonly, at an individual level of analysis, coauthorship data have been used as inputs to build up coauthorship (collaboration) networks, in the form of either whole-networks^7^,^10^,^13^,^14^,^15^,^16^, or in the form of personal networks (ego-neworks)^17^,^18^,^19^,^20^.

Ego-networks (personal networks) are made up of an *ego* (the focal actor), ego’s *alters* (actors ego has specific social relations with), *the relationships among alters* (alter-alter ties) *and between ego and alters*^21^. Personal network analysis focuses on the effects of social context (depicted as a specific pattern of ties) on individual characteristics, studying the social relations of individuals^22^. Generally, within personal network studies, two classes of variables have received closer attention: *network composition variables* (characteristics of the ego, of the alters, of ego-alter relationships, as well as of the alter-alter ties) and *network structure variables* (centrality measures such as degree, betweenness, closeness; aggregate variables such as density, core/periphery, number of components and of isolates, dyads, triads, efficiency, constraint etc.)^23^,^24^,^25^,^26^.

Personal networks have been of great interest in the study of various topics, such as: social support^27^, social protection^28^, health^29^, social capital^23^,^30^, searching a job^31^, migration^26^,^32^ etc. Interestingly, the studies employing a personal network analysis framework for the study of coauthorship are still scattered and sparse. A few studies stand up, reporting markedly contradictory results. For instance, it was investigated how scholarly performance associates with specific properties of personal coauthorship networks such as density, efficiency and constraint^17^. The corresponding results showed that scholars with more co-authors and with high score of betweenness centrality have higher g-index scores. Similar findings argued that sparse networks (with high betweenness centrality and average path length) exhibit higher levels of citations compared with dense networks^20^. However, contrastingly, a study conducted on a randomly selected sample of 238 authors from the Web of Science, indicated that, within personal coauthorship networks, betweenness centrality does not have any impact on the Hirsch-index score^19^. Furthermore, it was showed^19^ that personal networks’ size (number of alters) significantly accounts for 59% of the h-index scores of the egos. Additionally, compositional network variables, such as the mean of the tie (how many times two authors co-wrote together) and the mean of alters’ h-index score, even if statistically significant, do not have a major impact on the ego’s scientific performance (i.e. *R*^2^ = 0.02, respectively 0.01).

Due to the contradictions in the findings reported by the scant studies, further research work is evidently needed for clarification. Consequently, in this paper, *the first research objective was to assess whether personal coauthorship networks have a positive impact on researchers’ distribution of citations*. Unlike the prior related research studies, our intention was to extend the generality of the results, from collections of journals^17^ and samples of authors^19^,^20^ to populations of researchers. These populations were selected so as to comply with our second research objective: *to increase the level of knowledge on the influence coauthorship networks have on the research productivity of researchers from Eastern European countries*. We decided to have a closer look at the research populations embedded in Eastern European countries for at least two reasons. Firstly, these countries’ research production systems, generally, have received little attention in the area. Secondly, it is fruitful to understand why periodic evaluation reports systematically classify Eastern European countries as poor research performers. We are confident that learning about how coauthorship affects the impact of research productivity in these countries could contribute not only to the literature in the area, but also be in the benefit of policy makers. In addition, we add that we circumscribed our analysis to a research field wherein coauthorship has started only recently to emerge; precisely, sociology.

Scientific communities tend to express specific patterns of scientific collaboration and of propagation of citations. The so-called *Matthew effect*^33^ describes a process wherein prestigious scholars receive more recognition (including citations), compared to unknown ones. This process of self-reinforcing inequality, in which the *rich get richer and the poor get poorer*, is prevalent in the collaboration networks^34^. In the same vein, it is argued that large networks tend to follow a scale-free power-law distribution, according to which networks are permanently expanding by new nodes preferentially attaching to others already well-connected^35^. Presumably, collaboration networks hold this principle of preferential attachment. On these grounds, we can expect already established or highly visible researchers to have more opportunities of coauthorship and of benefits. It follows that a personal network, populated by alters exhibiting a superior scientific status vis-à-vis the ego, may be governed by the preferential attachment principle.

The *principle of homophily* is radically different from *the preferential attachment principle* or *the Matthew effect*. Homophily affects the composition of networks’ structure^36^. Precisely, homophily implies that a tie between similar people (as age, sex, occupation, social class, education etc.) has a higher probability of occurring (compared to the probability of a tie between dissimilar people). Building on the abundant literature empirically supporting the principle of homophily^36^,^37^,^38^, we assume similarity also governs the interactions between scholars and, consequently, the formation and composition of personal coauthorship networks. In this case, we operationally define homophily as visibility similarity measured as citation counts. Accordingly, we want to test for homophily or a “bird of a feather” effect. For this reason, our study looks at whether *the mean of alters’ citations has a positive impact on the number of citations of an ego* (Hypothesis 1).

Individual’s performance should also be addressed in association with the specific patterns of the networks they are embedded in refs 3,37. Within the field of social network analysis, one might discover various studies reporting relationships between specific structural properties and the individual performance. For instance, reports show positive relationships between structural holes (i.e. a node connects otherwise disconnected groups) and higher compensation, faster promotion, job evaluations and good ideas^39^. Evidence was presented that individual job performance is positively related, in organizational contexts, to centrality in advice networks and negatively related to centrality in hindrance networks (i.e. degree centrality: number of ties)^40^.

Network structure does matter not only in organizational settings^41^, but also in the field of sociology of science. In this area, *normalized betweenness*, *network size* and *density* have been employed as predictors for explaining variability in the *citation counts*. Previous work reported positive relationships between the individual performance (i.e. g-index scores, citation levels) of the egos and the betweenness centrality of their personal networks^17^,^20^. Moreover, it was showed a strong positive relationship between the network size and the h-index score of the egos^19^. Accordingly, we want to test for a “network structure effect” on the individual scientific performance. Building on these grounds, our study looks at whether *the normalized ego-betweenness score of a personal coauthorship network* (H2) and *the size of that network* (H3) *have a positive impact on the citations of an ego*. At the same time, we also assess whether *the density score of a personal coauthorship network has a negative impact on the citations of an ego* (H4).

For clarification reasons, *betweenness* equals the number of times an alter needs the ego to reach any other alter by the shortest path (geodesic distance)^24^. The *normalized* version simply divides the observed score of betweenness to its maximum value. This structural variable generally negatively correlates with the *network density* score which, in a binary personal network (wherein ties are either present or absent), is the proportion of all possible ties that are actually present. This is the motive for which, in our fourth hypothesis, we predicted a negative impact of *network density* on *ego’s citations*. Furthermore, the *size of a personal network* is simply the number of alters comprising a specific personal network^22^.

In order to test for the impact of the *mean of alters’ citations*, *normalized betweenness*, *network size* and *density* on the *citation counts* of a specific scholar, we employed a personal network analysis research design. Structural properties were measured at the level of personal coauthorship networks. The assessment of the predictors’ impact was carried out by fitting hierarchical regression models to the data. Additionally, special summarizing imageries were built, at the level of the personal networks, to scan for (potential) *preferential attachment* or *birds of a feather* effects.

Through all these, our study contributes to the understanding of how several types of effects (homophily, network structure effects, preferential attachment or Matthew effect) operate at the level of personal coauthorship structures, by influencing the distribution of citations. Differently uttered, *do the compositional and structural network factors have an impact on the citation counts?* By answering this question we provide clarification to the contradictory results reported by prior related research work. And in addition, our special focus on Poland (38.4 million population, 92,915 faculty members and 220,541 Web of Science publications), Romania (22.2 million population, 27,772 teaching and research faculty and 67,034 WofS publications) and Slovenia (2.1 million population, 5,742 teaching and research faculty, and 35,385 WofS publications) gives valuable insights for policy makers and other interested parts in understanding the deficit Eastern European countries manifest in terms of scientific impact and performance.

## Results

On average, Slovenian researchers have significantly more Web of Science (WoS) indexed journal publications (*M* = 6.1, *SD* = 5.4) compared with the Polish (*M* = 3.2, *SD* = 3.0) or the Romanian researchers (*M* = 1.3, *SD* = 3.0). Furthermore, all Polish and Slovenian researchers and only 41% of the Romanians (i.e. 120 out of 294) have WoS indexed journal publications. Also, the Slovenian scientific productivity exhibits higher levels of impact, in terms of citations (*M* = 31.7, *SD* = 50.2), as opposed to those of the Polish or Romanians (*M* = 11.2, *SD* = 64.1, and, respectively, *M* = 7.0, *SD* = 25.5). A similar tendency can be observed when looking at the mean scores of the researchers’ Hirsch index, with the Slovenians having a score approximately twice as much as the Romanians or Polish. Interestingly, not only the Slovenian egos, but also their alters display, on average, more citations (*M* = 2.4, *SD* = 1.3) than the co-authors of the academic researchers belonging to the other two populations. The Slovenian researchers, on average, have larger personal coauthorship networks. In these collaboration structures, they tend to facilitate the circulation of information among alters; generally lying on the shortest possible paths among alters (the average of normalized ego-betweenness is approaching 50). Romanian researchers possess denser personal networks wherein the normalized ego-betweenness is not very pronounced. The Polish networks, in terms of the average scores of the density and normalized ego-betweenness, are to be placed somewhere in between the Slovenian and Romanian personal networks.

Our study looks at whether ego’s publications, the mean score of alters’ citations, personal network size and the normalized ego-betweenness have a positive impact on the ego’s citations. Also, we assessed whether personal network density negatively impacts on the quality of an ego’s scientific outputs. In what it follows, we report the results of our hierarchical regression analysis (Table 1).

In the first step of the regression model, ego’s publications (our firstly introduced predictor) accounts for 26% (Poland), 32% (Romania) and 37% (Slovenia) in the variation of the ego’s citations (our dependent). The inclusion into the model of the *mean score of co-authors’ citations* (Step 2), as a second predictor, increases the explanation of the variation in ego’s citations with 17% (Poland), 27% (Romania) and 33% (Slovenia). Alternatively, a two-predictor model explains large amounts of the variation in the ego’s citations (70% for Slovenia, 59% for Romania and 43% for Poland). For the Romanian and Slovenian populations of researchers, the subsequent steps of the hierarchical regression model (i.e. Step 3 to 5) do not considerably improve the explanation of the variation in ego’s citations. Namely, the fluctuations of the *R*^*2*^ values, beginning with Step 3, tend to follow a rather flat slope. However, in the case of the Polish population, adding extra predictors in the succeeding steps of the model substantially increases the explanation of the dependent variable. For instance, the model in the last step (with all the predictors included) accounts for 57% of the variation in the outcome, compared to 43% (in Step 2). As a general remark, for each of the three populations, ignoring the ego’s publications (the control independent variable), the largest model improvement (Δ*R*^2^) in explaining the dependent variable’s variability is produced in Step 2 (i.e. with ego’s publications and the mean score of the co-authors’ citations as predictors). In other words, especially in the case of Romanians and Slovenians, personal network size and network structural features (normalized ego-betweenness and network density) did not consistently increase the multiple-correlation coefficient between the predictors and the dependent.

By and large, the predictors roughly manifest comparable behavior across the three populations. In Step 1, as ego’s publications increase with one standard deviation, the number of ego’s citations increases by 0.51 (Poland), 0.56 (Romania) and 0.61 (Slovenia) standard deviations. In Step 2, holding constant the effects of the ego’s publications, we observe that for one standard deviation increase of the mean score of the coauthors’ citations, the ego’s citations increase with 0.41 (Poland), 0.60 (Romania) and 0.58 (Slovenia).

For Romanian and Slovenian researchers, *ego’s publications* and *the mean score of the co-authors’ citations* remain, across all five steps of the model, the most important predictors. Moreover, the impact of compositional and structural network variables on ego’s citations is rather low. Namely, the variation in the ego’s citations due to one standard deviation increase of the number of co-authors, of normalized betweenness and of network density fluctuates between (only) −0.11 and 0.15 standard deviations. Contrary to the results obtained for the Romanian and Slovenian populations, in the case of Poland, predictors manifest a different behavior; especially, in the Steps 4 and 5 of the model. Specifically, the number of co-authors (the net size) has a greater positive effect on the ego’s citations compared to the mean score of co-authors’ citations. Contrary to our expectations, ego’s normalized betweenness negatively affects the dependent. As this structural predictor increases with one standard deviation, ego’s citations decreases with 0.92 standard deviations (while holding everything else constant).

**Figure 1:**
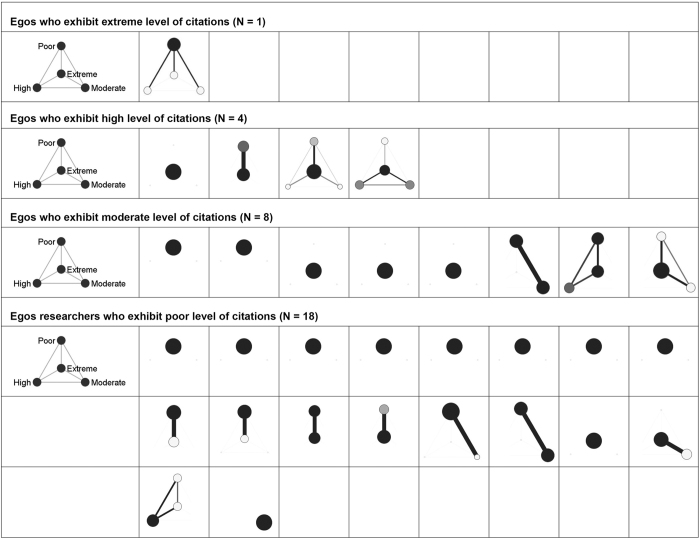
Visualizations of the clustered graphs for each of the 31 Polish academic researchers. The clustered graphs are the result of partitioning the personal networks on the criterion of alters’ citations. Node size reflects class size, darkness of a node reflects intra-class connectivity, width and darkness of a tie between nodes reflects the inter-class connectivity (tie count and tie-weight). Visualizations are split on four categories of Polish academic researchers based on their level of citations.

**Figure 2:**
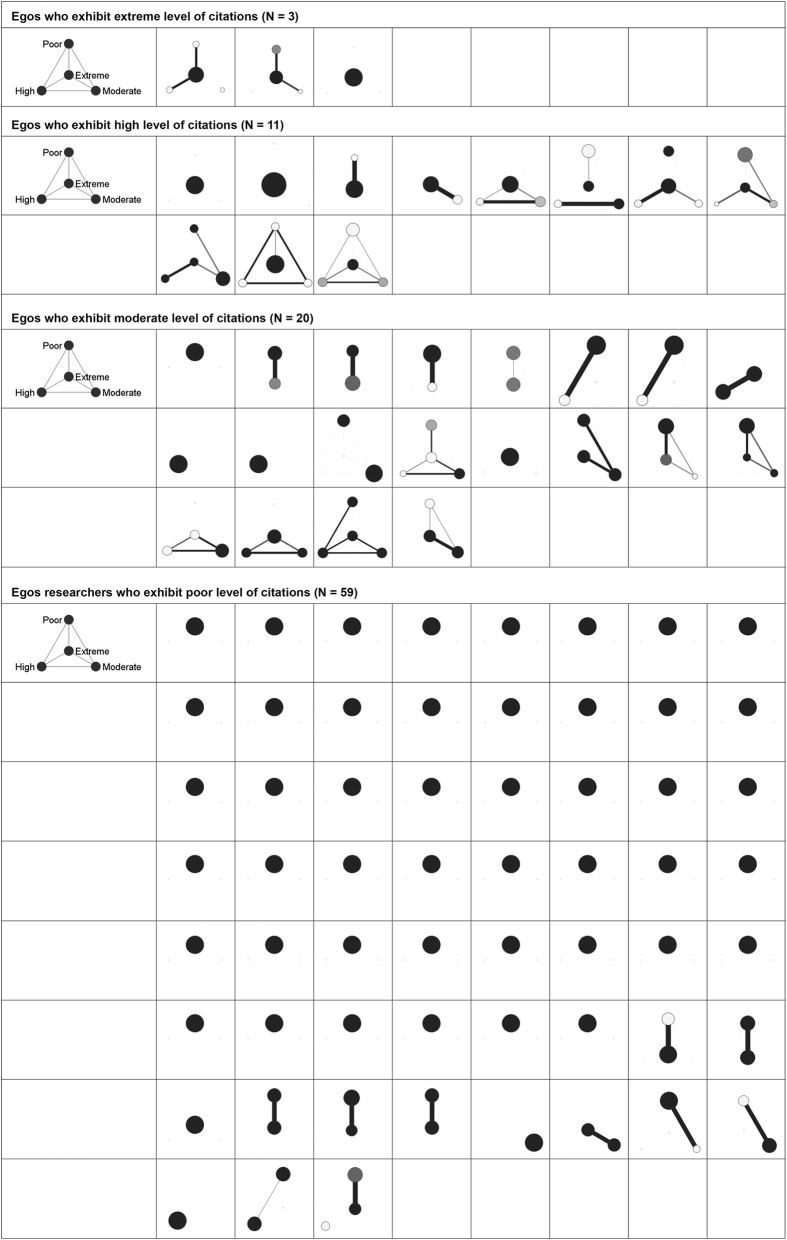
Visualizations of the clustered graphs for each of the 93 Romanian academic researchers. The clustered graphs are the result of partitioning the personal networks on the criterion of alters’ citations. Node size reflects class size, darkness of a node reflects intra-class connectivity, width and darkness of a tie between nodes reflects the inter-class connectivity (tie count and tie-weight). Visualizations are split on four categories of Romanian academic researchers based on their level of citations.

**Figure 3:**
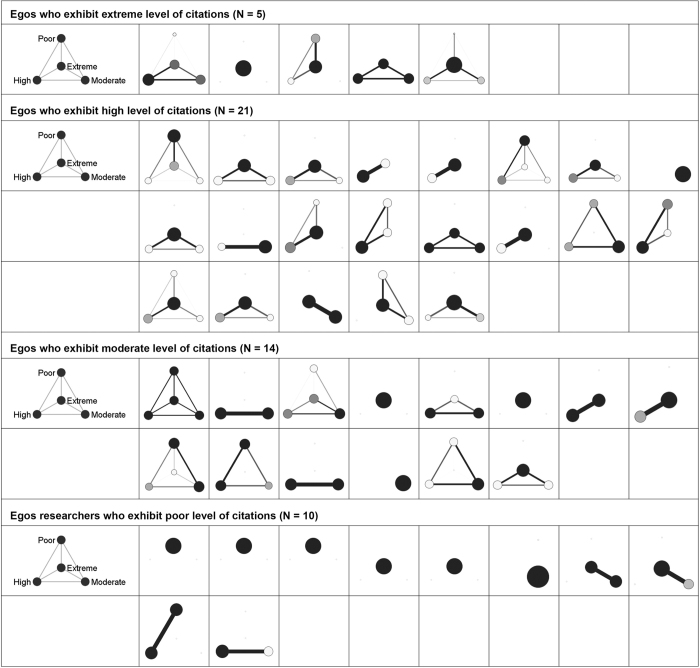
Visualizations of the clustered graphs for each of the 50 Slovenian academic researchers. The clustered graphs are the result of partitioning the personal networks on the criterion of alters’ citations. Node size reflects class size, darkness of a node reflects intra-class connectivity, width and darkness of a tie between nodes reflects the inter-class connectivity (tie count and tie-weight). Visualizations are split on four categories of Slovenian academic researchers based on their level of citations.

In the remainder of this section, we will present the personal coauthorship typologies as resulted after partitioning all the 174 personal coauthorship networks (Poland = 31, Romania = 93 and Slovenia = 50) into four classes of alters based on their citation counts. These clustered graphs (Figs 1–3) provide us with a micro-level qualitative insight that moves us a step ahead in understanding the fabric of the personal coauthorship patterns inside the three populations. For the purpose of a better inspection of the clustered graphs, we decided to split the egos (academic researchers) into four categories (Figs 1–3). Namely, using as a reference both egos’ and alters’ citation distributions inside each of the three populations, we devised the following categories: egos with *low* (zero and one citation), *moderate* (between two and ten citations), *high* (between eleven and 100 citations) and *extreme* (>100 citations) levels of citations. This four layered structure allows for a double comparison. On one hand, there is the possibility of comparing the structural patterns of the partitioned personal networks, inside each population, across the four categories of egos. On the other, there is the possibility of comparing the structural patterns of different types of egos across population of researchers.

**Figure 4:**
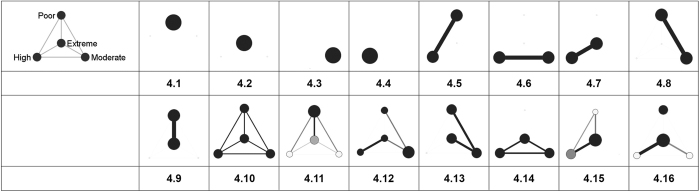
Some of the specific structural configurations within the personal coauthorship networks of the Polish, Romanian and Slovenian populations of researchers. The structural configurations are displayed through the use of clustered graph technique. Node size reflects class size, darkness of a node reflects intra-class connectivity, width and darkness of a tie between nodes reflects the inter-class connectivity (tie count and tie-weight). Visualizations are split on four categories of academic researchers based on their level of citations.

Examining the clustered graphs visualizations, there are several observations that can be brought forth. Firstly, the three populations of researchers (Polish, Romanian and Slovenian) are equally diverse in terms of the displayed structural configurations (some of these configurations are exhibited in Fig. 4). Secondly, one recurrent configuration among the researchers with poor level of citations is the one wherein the *poor* egos are embedded in personal networks of intensely inter-connected *poor* alters (Fig. 4.1). This type of configuration can also be distinguished, even if with a not comparable frequency, in the case of the other categories of researchers. As an example, some of the researchers of *extreme* level of citations are embedded in configurations wherein they are surrounded only by alters of extreme level of citations (Fig. 4.2). Equivalently, some of the researchers of *moderate* level of citations appear to be embedded in personal networks populated only by moderately cited alters (Fig. 4.3). In a different vein, radically different types of personal coauthorship networks are available. Namely, personal networks in which the egos are surrounded by alters with superior levels of citations. As an illustration, some of the researchers with low level of citations turn out to be part of structural configurations wherein they are adjacent to alters of moderate, high or/and extreme levels of citations (Fig. 4..9). Similar structural patterns can also be established in the case of both researchers of high level of citations (connected only to alters of extreme levels of citations; see Fig. 4.2) and of moderate level of citations (linked to alters of extreme and high levels of citations; see Fig. 4.7). Thirdly, some of the researchers (egos) appear to be embedded in networks marked by a high diversity of relational patterns (different inter-class tie patterns and intra-class connectivity) and populated with alters of different citation status (with distinct visibility/prestige profiles); some of these networks are displayed in Fig. 4..16. Fourthly, as a last remark, it is noteworthy there is no specific structural configuration to be attached to any of the four classes of egos (i.e. poorly, moderately, highly and extremely cited scholars; see Figs 1–3). Nevertheless, there is the notable exception of the Romanian and Polish researchers of poor level of citations. Apparently, for these researchers, the prevailing structural configuration is the one wherein they deploy personal networks populated by alters of *poor* levels of citations (Fig. 4.1).

## Discussion

Publication records show that Slovenian sociologists are more productive and more influential than the Polish and the Romanians. Generally, they appear to be embedded in larger-sized personal coauthorship networks, wherein they tend to facilitate the circulation of information among alters to a greater extent compared to their Romanian and Polish counterparts. Further, Romanian sociologists turn out to be embedded in smaller but denser personal networks.

For all the three populations, the number of publications and the prominence of the co-authors (in term of citations) jointly explain large shares of the variation in a researcher’s citations. The *mean score of the co-authors’ citations* has a substantive positive impact on *ego’s citations*, which provides evidence for our first research hypothesis. This result contradicts the prior findings^19^, according to which the mean alters’ h-index score does not have a major impact on ego’s h-index score.

Looking at our second hypothesis, we did not find evidence to support a consistent positive impact of normalized ego-betweenness on ego’s citations. In addition, our results were mixed. On one hand, we uncovered a positive effect of this predictor only for the Romanian population. But its size was rather small and its contribution to the explanatory power of the model was not substantial. On the other hand, contrary to our expectations, we found negative standardized beta coefficients in the case of Polish and Slovenian populations. Precisely, normalized ego-betweenness turned out to have a consistent (negative) effect on the ego’s citations only in the case of the Polish researchers. This evidence is inconsistent with the findings reported in the past^17^,^20^, according to which ego-betweenness positively affects citation levels. Our third hypothesis (according to which the network size has a positive impact on egos’ citation counts) was confirmed in the case of Polish (large-sized standardized beta coefficients) and Slovenian (small-sized coefficients) sociologists. These results partly support prior findings^19^, according to which network size positively impacts on ego’s h-index score. Nevertheless, for the Romanian population, the beta coefficients of the network size predictor oscillated around zero (with both positive and negative values across the steps of the hierarchical regression model). Our last hypothesis predicted a negative effect of personal network density on the ego’s citation count. The evidence was however mixed. Our expectation was confirmed for the Polish and Romanian populations (even if in the case of Romanians, we found a small-sized coefficient). As for the Slovenian population, our analysis elicited a positive effect.

All in all, our findings suggest that the mean score of co-authors’ citations generally has a positive impact on the ego’s citation counts. This might indicate the presence of homophily within each of the three populations of sociologists (i.e. birds of a feather effect). The other personal network predictors (network size, ego-betweenness and network density) substantially improve the explanation of individual research impact only in the case of the Polish academic researchers. Consequently, for Polish sociologists, working in large and cohesive research teams proves to be beneficial in terms of citations.

The homophily effect, signaled at the macro-level of the three populations of sociologists, is masked by the results brought forth at the micro-level analysis of the clustered graphs. Accordingly, after carefully examining the visual summarizations of the personal coauthorship networks, we found out a wide palette of structural configurations. This probably is a mark of the co-presence of various local and/or global practices of carrying out research and coauthorship. Specifically, we distinguished among three typologies of personal coauthorship networks. Firstly, there are clustered graphs displaying structures that are indicative of homophily (the birds of a feather effect). These personal networks show similarly cited egos and alters being embedded in the same coauthorship structures. Secondly, we identified personal coauthorship networks wherein the ego has either an inferior or a superior number of citations compared to her alters. On our view, this might be indicative of the preferential attachment principle (the Matthew effect). And, thirdly, there are personal coauthorship networks wherein egos and alters are defined by a large variability in terms of citations. This specific type of personal coauthorship network proved to be difficult to be assessed in terms of *birds of a feather* or *Matthew effect*.

Our study has at least two limitations. It lacks a longitudinal approach and also a qualitative-data oriented perspective. Even if effective for achieving the research objectives of our paper, the employment of a static approach in analyzing personal coauthorship networks proves to be rather limitative in its explanatory power. For this reason, our findings, inferred after carefully examining the collections of clustered graphs, are merely insights, signals or marks for the presence of the Matthew effect (the preferential attachment) or of birds of a feather effect (homophily). Furthermore, the results reported after conducting the hierarchical regression analysis are but screen shots corresponding to a specific moment in time. A way to improve the explanations about the citation cumulative processes and the development of personal coauthorship networks is to employ a longitudinal personal network analysis framework (a similar research mode was used in the study of migration^42^). Accordingly, our future intention is to approach coauthorship networks through the use of the Simulation Investigation for Empirical Network Analysis (SIENA) program. SIENA, generally, allows for the modeling of changing networks while taking into account the network structure, the individual attributes and the dyadic covariates^43^,^44^. Further, the second limitation in our study is represented by the lack of qualitative insights into the development across time of the personal coauthorship networks. We expect our future interviewing different categories of sociologists (classified on their citation counts) will allow us to validate or falsify our present findings, to enhance our understanding of the coauthorship practices in the three countries, and, also, to help us generate further research hypotheses.

We additionally suggest other possible future research directions. For instance, an enhancement in the understanding of how personal coauthorship networks affect the spread of citations can be brought forth: a) by including into analysis more actor attribute variables (such as age, gender or academic rank); b) by looking at the impact of non-domestic researchers moderated by levels of citations; c) by developing a multi-level approach for assessing the effects of department embeddedness and university impact.

Our paper displays at least two elements of originality. Firstly, to the best of our knowledge, our paper represents the first application of the visualization technique^45^ to the study of personal coauthorship networks. And, as a result, we believe the application of this technique appeared to be successful in determining personal coauthorship typologies. Secondly, it is the first study that analyzes collections of personal coauthorship networks collected from populations of researchers.

With everything considered, we would like to stress a few final remarks. Firstly, on average and from a macro-level perspective, the principle of homophily governs the personal coauthorship networks of the Polish, Romanian and Slovenian sociologists. In these countries, in the field of sociology, birds of a feather indeed write together. This entails at least one practical implication: the polarization of academic sociologists, with the highly, moderately and poorly cited separately co-authoring. Within this landscape, inequalities tend to augment and grow during time^34^. Additionally, any domestic public policy rewarding scientific performance will definitely reinforce with velocity the already existing inequalities among sociologists.

Secondly, at a micro-level perspective, the “birds of a feather effect” is masked, in all the three East European countries, by a variety of co-authoring practices. This might be, allegedly, the result of a multi-layered complex ensemble of factors: from institutional and organizational settings, collective and individual rational strategies to circumstantial and randomly displayed opportunities to co-author. Although this research has focused on sociologists from East European countries, our preliminary results using other data sources where sociologists also from other parts of Europe and the US are included, point to much the same phenomenon. We therefore expect our conclusions to hold also beyond the current geographical scope of study.

## Methods

We used empirical data collected from three populations of *academic researchers* (i.e. full-time embedded in university departments of sociology), from Poland (*N* = 55), Romania (*N* = 294) and Slovenia (*N* = 58). Specifically, the building principle for each of the three populations was that of *structural equivalence*. Two actors are considered structurally equivalent if they have identical relations with all the other individuals in a population^46^. Satisfying the condition of structural equivalence, we wanted to ensure proper significance and comparability of the results. Consequently, we selected from each of the three countries only academic researchers working in university departments of *sociology*. We assumed all individuals in each of these populations confront with identical institutional pressures (funding opportunities, academic career regulations, incentives for publications etc.). Further, that they possess the same configuration of professional relationships, and undertake roughly similar teaching and research activities.

We generated the three populations, using the following steps. Firstly, we identified all the university departments of sociology, in each of the three countries. Secondly, we inspected the official (institutional) webpages of those departments and listed the names of all the full-time working affiliated academic researchers (i.e. only members granted tenure). Thirdly, we used the names listed in advanced as to collect research productivity metrics from the Thomson Reuters’ Web of Science electronic archive (WoS). As a result, a bibliometric record was produced for every individual in the three populations. That record was afterwards used, in a fourth step, to generate lists of co-authors (where available). In a last step, for every co-author a bibliometric record was also produced.

Eventually, we assembled a dataset containing information about both egos (the academic researchers) and alters (the co-authors of the academic researchers). On a later stage, that dataset was supplemented with structural measurements conducted on the resulted personal coauthorship networks. The information referred to both our dependent variable (ego’s citation counts) and independent variables (number of publications, mean score of alters’ citations, normalized ego-betweenness, network density and size). Additionally, for future analyses, other variables were measured: gender, nationality, university department affiliation, number of co-authors, Hirsch-index score, alters’ maximum Hirsch-index score, the author per publication ratio. It should be mentioned that the data were collected as of January, 2016.

We did not employed special algorithms for treating the problems of disambiguation, as we considered them inappropriate for the purposes of our study. Instead, we decided to replicate a different procedure^19^. Accordingly, a team of reviewers, composed of undergraduates, was trained and appointed to disambiguate the authors. Before generating the bibliometric records (of authors and co-authors), guidelines had been devised and provided to the team of reviewers. Affiliations, publication records and vitae, for every author and co-author, were cross-referenced (where necessary) as to ensure accuracy and consistency.

In this study, we employed a personal network analysis research design^47^,^48^. In our personal networks of coauthorship ties, as a rule, the academic researchers from the three populations were appointed as egos, and their co-authors, as alters. We worked with first-order personal networks which means we represented not only the coauthorship ties between an ego and her alters (ego-alter ties), but also the coauthorship ties among alters (alter-alter ties)^49^. Technically, the personal networks were built using the Egocentric Network Study Software - EgoNet^50^. Afterwards, the resulted outputs (the collections of personal networks) were transferred to UCINET^51^ for structural measurements (e.g. the computing of scores corresponding to: normalized betweenness, network size and density).

SPSS built hierarchical multiple-regression models^52^ were used to test for the hypothesized effects (“bird of a feather” and “network structure” effects); in other words, to account for the ego’s citations (the dependent variable). We devised the hierarchical models, after establishing the substantial theoretical importance of each predictor (i.e. predictors were selected based on past research work; see the *Introduction* section of this paper). Predictors known from previous related literature to have higher impact in predicting the dependent variable were introduced first. Accordingly, *ego’s publications* variable was the firstly entered predictor. Secondly, we entered *the mean score of alters’ citations*. In the construction of this predictor, we filtered out the citations alters had in common with their egos (i.e. to avoid inflating the correlation between ego’s citations and the mean score of alters’ citations). Thirdly, we entered into the regression *the number of co-authors* (network size). The structural predictors (*normalized ego-betweenness and density*) were introduced in the end. As an aside, we chose to use the logs of the original raw values (i.e. log(*Y*)), as to increase linearity and handle positive skewed distributions. We added the constant *0*.*0001* to all variables before the log transformations; as to prevent the problems non-positive scores generally create).

The visual variables (e.g. position, shape, color, connections, labels etc.) are a special class of variables in the analysis of personal networks. Visualizations are extremely effective and practical for the identifying and construction of typologies of personal networks, especially in the case of analyzing important amounts of information^26^. In this line, it was developed *the clustered graph technique*, a visual methodology for standardizing and comparing collections of personal networks^42^,^45^,^53^. Particularly, this method visually summarizes personal networks on which a clustering of the vertices is given^45^. Normally, every researcher chooses the relevant variables for the partitioning (clustering) of the data, based on the objectives of her research project. As a result, the clustered graph technique provides a simple version of an initial personal network (or collections of personal networks), retaining characteristics important for the analysis (e.g. density of the classes or of the relationships among classes).

**Figure 5:**
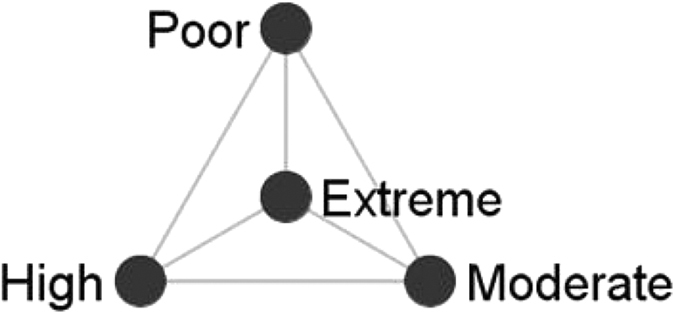
Visual summarization for the personal coauthorship network of a researcher, using the clustered graph technique. The researcher (the ego) is excluded from the visualization. Alters are partitioned into four classes on the criterion of citation counts (i.e. alters with *poor*, *moderate*, *high* and, respectively, *extreme* level of citations). Node size reflects class size while node color indicates the intra-class connectivity (the darker the color, the higher the connectivity among alters within that specific class). Tie color and width illustrate the inter-class connectivity (the darker/the thicker the line, the higher the inter-class connectivity).

We used this visual technique to scan for the potential presence of the “birds of a feather” effect and of the Matthew effect (the preferential attachment principle) at the level of the structural configurations of coauthorship ties. According to the suggestions available in the literature, we built class-level networks in two steps^45^. Firstly, we defined four actor classes, by partitioning the set of alters on the criterion of citation counts (i.e. how many citations a specific alter has received through time). Using the quartiles of alters’ citation distribution, we devised: the class of alters with a *poor* level of citations (first quartile), the class of alters with a *moderate* level of citations (second quartile), the class of alters with a *high* level of citation (third quartile) and the class of alters with an extreme level of citations (outliers). Secondly, we used visual variables to represent the size of the classes and the connectivity among and within the classes. The node size reflected the class size (i.e. number of alters in each class), the darkness of a node reflected the intra-class connectivity (as node grows darker, connectivity increases), and the width and darkness of a tie between two nodes reflected the inter-class connectivity (as tie grows thicker and darker, connectivity increases). Figure 5 illustrates the layout of these classes. Technically, the clustered graphs were created using *visone* software^54^. The personal coauthorship network data, previously built within EgoNet^50^, were loaded into visone, following prior described methodology^45^. Using the visone routines, we were able to cluster, aggregate and visualize the three collections of personal coauthorship networks (corresponding to populations of academic researchers of Poland, Romania and Slovenia). It should be noted that the clustered graphs generally are the visual summarization of original (initial) personal networks wherein the ego is not represented (an analysis of different forms of personal network visualizations is available in the area^55^). Excluding the ego from the visualization of her personal network provides a special representation of the structural pattern^56^.

## Additional Information

**How to cite this article**: Hâncean, M.-G. and Perc, M. Homophily in coauthorship networks of East European sociologists. *Sci. Rep.* **6**, 36152; doi: 10.1038/srep36152 (2016).

**Publisher’s note:** Springer Nature remains neutral with regard to jurisdictional claims in published maps and institutional affiliations.

## Figures and Tables

**Table 1:** The results of a five step hierarchical regression model accounting for scholars’ citation counts.

	Variable	Step 1	Step 2	Step 3	Step 4	Step 5
	*Poland*					
1	Ego’s publications (ln)	0.51	0.53	0.50	0.90	1.02
2	Mean score of alters’ citations (ln)		0.41	0.36	0.27	0.37
3	Ego’s coauthors (ln)			0.16	0.54	0.78
4	Ego’s normalized betweenness (ln)				−0.65	−0.92
5	Personal network density (ln)					−0.29
	* R*^2^	0.26	0.43	0.45	0.53	0.57
	Δ*R*^2^	0.26	0.17	0.02	0.08	0.04
	*Romania*					
1	Ego’s publications (ln)	0.56	0.28	0.26	0.21	0.21
2	Mean score of alters’ citations (ln)		0.60	0.59	0.56	0.57
3	Ego’s coauthors (ln)			0.03	−0.04	−0.02
4	Ego’s normalized betweenness (ln)				0.15	0.14
5	Personal network density (ln)					−0.02
	* R*^2^	0.32	0.59	0.59	0.60	0.60
	Δ*R*^2^	0.32	0.27	0.00	0.01	0.00
	*Slovenia*					
1	Ego’s publications (ln)	0.61	0.50	0.47	0.52	0.50
2	Mean score of alters’ citations (ln)		0.58	0.57	0.58	0.60
3	Ego’s coauthors (ln)			0.08	0.12	0.08
4	Ego’s normalized betweenness (ln)				−0.11	−0.07
5	Personal network density (ln)					0.11
	* R*^2^	0.37	0.70	0.70	0.71	0.72
	Δ*R*^2^	0.37	0.33	0.00	0.01	0.01

The model was fit to empirical data corresponding to three populations of academic researchers embedded in university departments of sociology (Poland, Romania, and Slovenia). Variables were log-transformed before conducting the analysis. Standardized coefficients (beta) are displayed.
